# Regenerative Effects of Mesenchymal Stem Cells: Contribution of Muse Cells, a Novel Pluripotent Stem Cell Type that Resides in Mesenchymal Cells

**DOI:** 10.3390/cells1041045

**Published:** 2012-11-08

**Authors:** Shohei Wakao, Yasumasa Kuroda, Fumitaka Ogura, Taeko Shigemoto, Mari Dezawa

**Affiliations:** 1 Department of Stem Cell Biology and Histology, Tohoku University Graduate School of Medicine, Sendai, 980-8575, Japan; Email: wakao@med.tohoku.ac.jp (S.W.); f.ogura@med.tohoku.ac.jp (F.O.); tshigemoto@med.tohoku.ac.jp (T.S.); 2 Department of Anatomy and Anthropology, Tohoku University Graduate School of Medicine, Sendai, 980-8575, Japan; Email: y-kuroda@med.tohoku.ac.jp

**Keywords:** pluripotent stem cells, mesenchymal stem cells, transdifferentiation, tissue repair, cell therapy

## Abstract

Mesenchymal stem cells (MSCs) are easily accessible and safe for regenerative medicine. MSCs exert trophic, immunomodulatory, anti-apoptotic, and tissue regeneration effects in a variety of tissues and organs, but their entity remains an enigma. Because MSCs are generally harvested from mesenchymal tissues, such as bone marrow, adipose tissue, or umbilical cord as adherent cells, MSCs comprise crude cell populations and are heterogeneous. The specific cells responsible for each effect have not been clarified. The most interesting property of MSCs is that, despite being adult stem cells that belong to the mesenchymal tissue lineage, they are able to differentiate into a broad spectrum of cells beyond the boundary of mesodermal lineage cells into ectodermal or endodermal lineages, and repair tissues. The broad spectrum of differentiation ability and tissue-repairing effects of MSCs might be mediated in part by the presence of a novel pluripotent stem cell type recently found in adult human mesenchymal tissues, termed multilineage-differentiating stress enduring (Muse) cells. Here we review recently updated studies of the regenerative effects of MSCs and discuss their potential in regenerative medicine.

## 1. Introduction

Mesenchymal stem cells **(**MSCs) are cells that reside in tissues such as bone marrow, fat tissue, dermis, and the umbilical cord, and are useful for cell-based therapy in humans because of their low risk of tumorigenesis [1,2]. MSCs have pleiotropic actions; not only do they exert trophic and anti-inflammatory effects on damaged tissues by producing a variety of trophic factors and cytokines, but they also modulate immunologic reactions, which is the basis for their application in graft-versus-host disease [3,4,5]. 

Besides the mechanisms underlying the trophic, anti-inflammatory, and immunomodulatory effects, another important action of MSCs that has been debated over the past decade is the broad spectrum of differentiation beyond the boundaries of tissue stem cells. Generally, tissue stem cells generate the cell types of the tissue in which they reside, and thus the range of their differentiation capabilities is usually limited. For example, hematopoietic stem cells generate blood cells and neural stem cells generate neurons, astrocytes, and oligodendrocytes [6,7]. MSCs belong to the mesodermal lineage, so it would be reasonable that they differentiate into cells of the same mesodermal lineage, such as osteocytes, cartilage cells, and adipocytes [8,9,10,11], and other mesodermal lineage cells, such as skeletal muscle cells, endothelial cells, and cardiomyocytes [8,9,10,11]. Interestingly, however, MSCs also differentiate into the other lineages, endodermal and ectodermal cells, such as hepatocytes, insulin-producing cells, neuronal cells, and peripheral glial cells [12,13,14,15,16]. Such a broad spectrum of differentiation has been observed in *in vitro* experiments using cytokine induction and/or gene introduction. MSCs also spontaneously differentiate into mesodermal, ectodermal, or endodermal cells with a very low frequency *in vivo.* When transplanted, these cells home to the damaged site and differentiate into cardiomyocytes (mesodermal), hepatocytes (endodermal), and keratinocytes (ectodermal) according to the local microenvironment they integrated and contribute to tissue repair [17,18,19]*.*

Based on these reports*,* it has been speculated that MSCs contain cells resembling pluripotent stem cells that also work as ‘repair cells’* in vivo*, but because of a lower ratio of triploblastic differentiation, such putative cells are considered to comprise a very small subpopulation of MSCs. Recently, adult human mesenchymal cells have been reported to contain a novel type of pluripotent stem cell population that may explain the triploblastic differentiation and tissue repair effect observed in MSCs [20]. In this review, the unique properties of MSCs and their great possibility for regenerative medicine are discussed.

## 2. MSC Heterogeneity

MSCs are usually harvested just as adherent cells from mesenchymal tissues, such as the dermis, bone marrow, adipose tissue, and umbilical cord. Due to this unsophisticated simple method of collection, MSCs are seen as crude cell populations comprising a heterogeneous population. The individual populations comprising mesenchymal cells often differ with regard to origin, phenotype, and differentiation state [21]. For simplicity, however, such crude cells are called MSCs, even though a robust characterization of their stemness is lacking.

Dermal fibroblasts are one type of the MSCs that are usually collected from adherent dermal cell cultures, but the so-called fibroblasts are not a single cell population because the dermis comprises various cell types, such as fibroblasts (the major component of the connective tissue), blood vessel-associated cells (endothelial cells and pericytes), sensory nerve-related glial cells (Schwann cells), as well as several types of stem or progenitor cells, such as skin-derived precursors, neural crest-derived stem cells, melanoblasts, perivascular cells, endothelial progenitors, and adipose-derived stem cells [22,23,24,25,26,27,28,29]. In fact, primary cultured dermal cells contain cells positive for CD117 (a marker for melanoblasts), CD146 (perivascular cells and adipose-derived stem cells), CD271 (neural crest-derived stem cells), Snai1 (skin-derived precursors), and Slug (skin-derived precursors) [3]. 

This cell population heterogeneity is also found in another mesenchymal cell type, bone marrow-derived mesenchymal cells. These cells are often called bone marrow MSCs (BM-MSCs) and are also usually collected as adherent cells from bone marrow aspirates and are thus heterogeneous. As reported by Pittenger *et al.* [8], BM-MSCs are positive for mesenchymal markers, but the marker content and expression ratios differ among batches. 

The definition of a ‘stem cell’ requires that the cells possess two properties, self-renewal (the ability to renew themselves through mitotic cell division) and potency (ability to differentiate into a diverse range of specialized cell types) [30]. Potency specifies the differentiation potential of the stem cell; pluripotent stem cells are defined as cells that can differentiate into cells of either ectodermal, endodermal, or mesodermal lineage, and multipotent stem cells are defined as those that can differentiate into a number of cells, mostly those of a related family of cells that belong to the same cell lineage such as in the case of differentiation of MSCs into osteocytes, adipocytes, and chondrocytes [30]. To be precise, stem cells must meet these requirements at a single cell level, as seen in the characterization of neural stem cells: sphere formation and differentiation into neurons and glial cells. In the case of MSCs, however, the heterogeneity makes it difficult to appropriately verify putative rare pluripotent stem cells that might be responsible for triploblastic differentiation. From that standpoint, the differentiation ability of MSCs has remained an enigma.

## 3. Controversy over Pluripotency of Mesenchymal Cells

Over the past decade, it has been argued whether MSCs could have pluripotency characteristics. Verfaillie *et al.* described that MSCs derived from adult bone marrow, which they named multipotent adult progenitor cells (MAPC). MAPCs could also be considered a pluripotent stem cell type because they can be differentiated into cells representative of all three germ layers [31]. Because other laboratories have not been able to produce MAPCs, however, their existence has been questioned. Ratajczak *et al.* reported that a population of very small embryonic-like cells, named VSEL cells, expressing the known embryonic stem (ES) cell markers Oct-4, Nanog, and Rex-1, are able to differentiate into cardiac (mesodermal), neural (ectodermal), and pancreatic (endodermal) cells and therefore are pluripotent stem cells [32], but the existence of VSEL cells has also recently been questioned by another group [33]. While the reports of pluripotent cells are exciting and suggest the potential pluripotency of MSCs, their existence is uncertain due to insufficient identification of specific convincing markers for MAPCs or VSEL cells and the lack of reproducibility between different labs.

As mentioned above, the definition of ‘pluripotent stem cells’ applies both to triploblastic differentiation and self-renewal. In addition to the above two properties that mimic normal development, however, definition of pluripotency often includes germ line-transmitting chimeras and/or teratomas [30,34]. This is typically observed with ES cells and induced pluripotent stem (iPS) cells, while another type of pluripotent stem cell type, epiblast stem cells, does not form teratomas under certain circumstances [35].

The argument of MSC pluripotency has been argued because MSC do not produce the germ line-transmitting chimeras and/or teratomas in question. MSCs indeed show triploblastic differentiation both *in vitro* and *in vivo*, and in this regard they are often called pluripotent. MSCs do not contribute to teratoma formation nor germ line transmitting chimeras, however, and thus MSCs are sometimes described as 'multipotent' and not pluripotent [8]. It is understandable that MSCs, which normally reside in mesenchymal tissues, are not tumorigenic and thus do not from teratomas when transplanted *in vivo.* There may be fundamental differences between MSCs and cells that contribute to germ line-transmitting chimeras, such as ES cells. The term 'multipotency', however, may not be adequate to describe their triploblastic differentiation ability. Overall, ‘self-renewal’ and ‘triploblastic differentiation’ could be considered essential and common requirements for all kinds of pluripotent stem cells, and these two properties are sufficiently comprehensive and practical to represent the high differentiation ability of MSCs rather than setting limits by including generation of germ line-transmitting chimeras and/or teratoma formation abilities. More importantly, if cells that are pluripotent but do not form tumors can be obtained from normal human tissues, this will be beneficial for cell-based therapy. 

## 4. Mesenchymal Cells Contain Pluripotent Stem Cells

A novel pluripotent stem cell type, multilineage-differentiating stress enduring (Muse) cells, has recently been identified in adult human mesenchymal tissues [20]. Muse cells are unique because they show pluripotency aspects such as the expression of pluripotency markers, self-renewal ability and triploblastic differentiation, while at the same time they also demonstrate the characteristics of mesenchymal cells [20]. In other words, they are double-edged, both pluripotent and mesenchymal cell-like. 

Muse cells were found initially as stress-tolerant cells [20]. Tissue stem cells are normally dormant and not active, but once the tissue is damaged or exposed to stress, they are activated to start proliferating, differentiating, and contributing to tissue repair. For example, neural stem cells that are located in the brain are normally inactive, but following stroke, those stem cells enter into the cell cycle and generate neural cells, including neurons [36]. Muse cells are a kind of stem cell that is stress tolerant.

**Figure 1:**
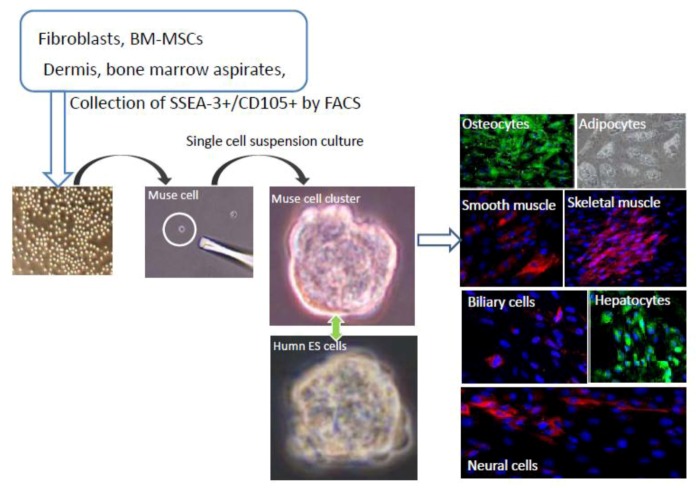
Isolation and characterization of Muse cells.

When mesenchymal cells, such as human BM-MSCs or dermal fibroblasts, are cultured for longer than overnight under stress-inducing conditions, *i.e.*, trypsin incubation or low nutrition, the large majority of mesenchymal cells dies out and only a small number of cells survive. When those surviving cells are cultured in a single cell-suspension culture, they form clusters that are very similar to the embryoid bodies formed by human ES cells that express pluripotency markers, show triploblastic differentiation, and self-renewal ability (Figure 1). To collectively represent the properties of those cells, they were named multilineage-differentiating stress enduring cells [20].

Muse cells can be collected from cultured mesenchymal cells (fibroblasts or BM-MSCs) and mesenchymal tissues (dermis, and bone marrow aspirates) as cells double positive for SSEA-3 and CD105. Collected Muse cells express SSEA-3 on their surface, similar to human embryonic stem (ES) cells. After isolating Muse cells by fluorescence-activated cell sorting, single Muse cells cultured in suspension (single cell-suspension culture) generate characteristic clusters that are very similar to the embryoid bodies formed by human ES cells. When the cell clusters are transferred onto gelatin culture and spontaneous differentiation is induced, cells with endodermal- (hepatocytes; α-fetoprotein+ and biliary cells; cytokeratin7+), ectodermal- (neuronal cells; neurofilament+), and mesodermal- (osteocytes; osteocalcin+, adipocytes; lipid droplets+, smooth muscle cells; smooth muscle actin+, skeletal muscle cells; desmin+) lineage are observed. (See Figure 1, pictures adapted with permission from Y. Kuroda *et al.* (2010). 2010 The National Academy of Science, and with permission from Wakao *et al*. (2011). 2011 The National Academy of Science.) [3,20]. 

A specific marker for Muse cells was investigated by comparing the expression pattern of cells before and after long-term stress incubation. Among many candidate cell surface markers, a positive ratio for SSEA-3, a well-known marker for undifferentiated human ES cells, drastically increases after stress incubation. When human fibroblasts and BM-MSCs are separated into SSEA-3-positive and SSEA-3-negative populations and subjected to single cell-suspension culture, only the SSEA-3 positive cells generate the clusters mentioned above, indicating that Muse cells are SSEA-3-positive cells [20]. Muse cells are further clarified to express mesenchymal markers, such as CD29, CD90, and CD105, so that Muse cells can be identified as cells double-positive for mesenchymal and pluripotency markers (Figure 1) [3]. 

Muse cells are double-edged, not only in the uniqueness of their surface marker expression profile, but also in their behavior and other properties. In adherent culture, they appear the same as the general population of mesenchymal cells, such as fibroblasts, but when they are transferred to a single cell-suspension culture, the cells proliferate and form cell clusters that resemble ES embryoid bodies (Figure 1). Such single cell-derived Muse cell clusters are not only similar to pluripotent stem cells like ES cells in their appearance, but also in their positivity for alkaline phosphatase as well as for the pluripotency markers Nanog, Oct3/4, and Sox2. Most importantly, they differentiate into endodermal-, ectodermal-, and mesodermal-lineage cells when transferred to gelatin cultures, indicating that single Muse cells are able to generate cells representative of all three germ layers (Figure 1). Muse cells are also able to self-renew while maintaining a normal karyotype [3,20]. 

As mentioned above, the existence of pluripotent cells in MSCs has long been suggested, but to date there have been no reports clearly demonstrating self-renewal and triploblastic differentiation at a single cell level, so the pluripotency among MSCs remained controversial [37,38]. Most importantly, single Muse cells are able to generate osteocytes, adipocytes, chondrocytes, skeletal muscle cells, smooth muscle cells (mesodermal-lineage); neuronal cells, epidermal cells (ectodermal-lineage); and hepatocytes, and biliary cells (endodermal-lineage cells) *in vitro*, and keep self-renewing, so that they are considered pluripotent stem cells (Figure 1). When Muse cells spontaneously differentiate (*i.e.*, without trophic factors), the mesodermal-lineage differentiation percentage is slightly higher (10%~15%) than ectodermal (3%~4%) or endodermal (3%~4%)-lineage-differentiated cells that cross over the oligolineage boundaries between germ layers [3,20]. 

## 5. Muse Cells Are Directly Reprogrammed into Desired Cells by Induction

While Muse cells spontaneously differentiate into mesodermal-, ectodermal-, and endodermal-lineage cells, their differentiation ratio is not very high. When Muse cells are treated with a certain combination of cytokines and trophic factors, more than 90% of the cells can be directed to differentiate into purposive cells. For example, when Muse cells are treated with hepatocyte growth factor, fibroblast growth factor 4, and dexamethasone in insulin-transferrin-selenite medium, ~90% of the cells become positive for alpha-fetoprotein and human albumin, hepatocyte markers, within 4 weeks. Similarly, ~90% of Muse cells treated with Neurobasal medium with B-27 supplement, basic fibroblast growth factor, and EGF differentiate into MAP-2- or neurofilament-positive cells by neuronal induction; namely, generating neurospheres, followed by differentiation into neuronal cells when treated with basic fibroblast growth factor and brain-derived neurotrophic factor. In osteocyte or adipocyte induction medium, ~98% of Muse cells differentiate into cells positive for osteocalcin or oil-red, respectively. In this manner, desired cells either of mesodermal-, ectodermal- or endodermal-lineage cells can be efficiently obtained from Muse cells depending on the induction treatment. More importantly, none of the above-mentioned differentiations require the introduction of exogenous genes, so that Muse cells produce the desired cells with lower risks [3]. 

## 6. Muse Cells are Different from Known Stem Cells in the Mesenchymal Tissues, Dermis, and Bone Marrow

While Muse cells are found in the bone marrow and dermis, these tissues also contain several kinds of stem cells. Bone marrow contains several stem cell types, including BM-MSCs, hematopoietic lineage cells, and endothelial cells [19]. To determine the localization of Muse cells within these different cell types, mononucleated cells were isolated from bone marrow aspirate and separated into hematopoietic (CD34 and CD117 double-positive) and BM-MSC (CD105-positive) fractions, as well as the rest of the cells (negative for CD34, CD117, and CD105). The majority of Muse cells that form human ES cell-like clusters and show self-renewal and triploblastic differentiation belong to the CD105[+] bone marrow-population [3]. 

The adult human dermis contains several types of stem or progenitor cells as described above [22,23,24,25,26,27,28,29]. Muse cells are all negative for NG2 (a marker for perivascular cells), CD34 (endothelial progenitors and adipose-derived stem cells), von Willebrand factor (endothelial progenitors), CD31 (endothelial progenitors), CD117 (melanoblasts), CD146 (perivascular cells and adipose-derived stem cells), CD271 (neural crest-derived stem cells), Sox10 (neural crest-derived stem cells), Snai1 (marker for skin-derived precursors), Slug (skin-derived precursors), Tyrp1 (melanoblasts), and Dct (melanoblasts), suggesting that Muse cells are a different cell type from known stem or progenitor cells found in the adult human dermis [3]. Thus, Muse cells are a novel type of stem cell found in the bone marrow and dermis. 

## 7. The Localization and Ratio of Muse Cells *in Vivo*

**Figure 2:**
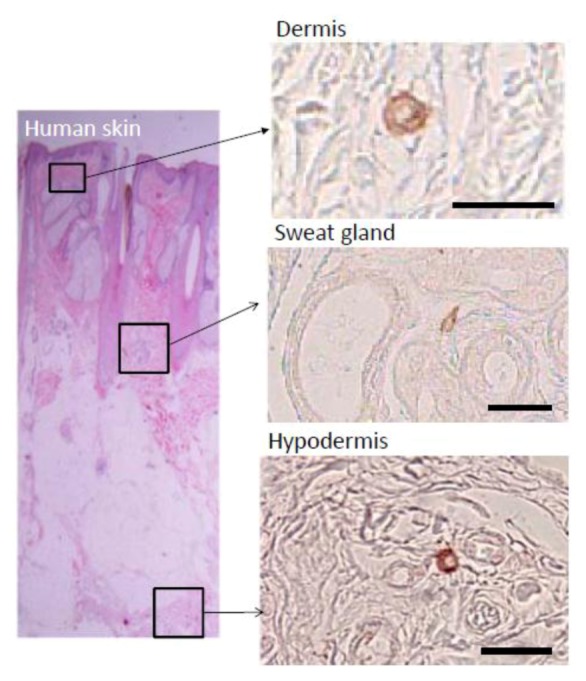
Localization of Muse cells in adult human skin.

Histologically, Muse cells, detected as SSEA-3-positive cells, locate sparsely in the connective tissues of organs. In the human dermis, Muse cells locate in the connective tissues distributed in the dermis and hypodermis, and in most cases they are scattered sparsely in the connective tissue and do not associate with particular structures such as blood vessels or dermal papilla (Figure 2) [3]. Other than the dermis, Muse cells are detected in the connective tissue of other tissues, however; rather, mesenchymal tissues such as bone marrow and skin are realistic sources for obtaining Muse cells. 

Muse cells labeled by SSEA-3 are sparsely observed in the connective tissue of the dermis, sweat glands, and hypodermis. (Figure 2, pictures adapted with permission from Wakao *et al*. (2011). 2011 The National Academy of Science.) [3].

In the case of bone marrow aspirate, SSEA-3/CD105 double-positive Muse cells are contained at the ratio of 0.03%, namely, 1 in 3000 mononucleated cells. The proliferation speed of Muse cells is ~1.3 d/cell division, so that 10 ml fresh bone marrow aspirate yields approximately one million Muse cells by 10 days [20]. 

Cultured mesenchymal cells such as human dermal fibroblasts and BM-MSCs are another realistic source for Muse cells. In fibroblasts and BM-MSCs, the ratio of Muse cells ranges from around 1% to at most 5%~6%, however the ratio and quality of Muse cells are altered by handling and the number of subcultures. 

## 8. Muse Cells are Non-Tumorigenic Pluripotent Stem Cells

Investigation of the expression of the genes related to pluripotency, such as Nanog, Oct3/4, and Sox2, in Muse cells revealed a ‘repertoire’ of expressed factors similar to that of ES and iPS cells, while the ‘expression level’ of those factors is very low in Muse cells compared to ES and iPS cells. ES cells and iPS cells have high levels of telomerase activity as well as high expression levels of genes related to cell-cycle progression, whereas Muse cells have low levels of both of these activities, the same level as in naive fibroblasts [3]. 

**Figure 3:**
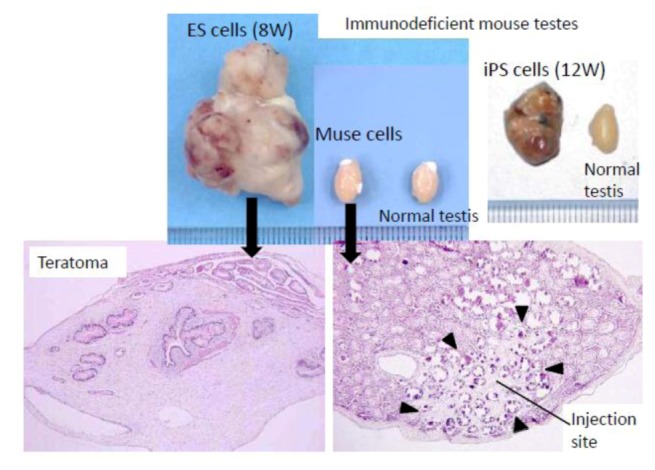
Non-tumorigenic properties of Muse cells.

ES and iPS cells form teratomas when transplanted *in vivo*. For example, when those cells are transplanted into the testes of immunodeficient mice, teratomas form within 8 to 12 weeks (Figure 3). In contrast to these pluripotent stem cells, however, Muse cells do not develop into teratomas *in vivo*. None of the Muse cell-transplanted immunodeficient mouse testes formed teratomas even after 6 months (Figure 3) [3,20]. The non-tumorigenicity of Muse cells is consistent with the fact that they reside in normal adult mesenchymal tissue.

When embryonic stem (ES) or induced pluripotent stem (iPS) cells were infused into immunodeficient mice (SCID mice) testes, they formed teratomas within 8 to 12 weeks. In contrast, none of the Muse cell-transplanted testes generated teratomas. (Figure 3, pictures adapted with permission from Y. Kuroda *et al*. (2010). 2010 The National Academy of Science, and with permission from Wakao *et al*. (2011). 2011 The National Academy of Science.) [3,20].

## 9. Tissue Repairing Function of Muse Cells *in Vivo*

When infused *in vivo*, a small number of the cells among MSCs migrate to and integrate into the damaged site. They differentiate into tissue-specific cells according to the microenvironment they homed and contribute to tissue repair in various kinds of organs and tissues, so that ‘repairing cells’, the cells that repair tissues across mesodermal, ectodermal and endodermal lineages *in vivo*, are assumed to exist among MSCs. Because of their very low frequency, however, the cells among MSCs responsible for this phenomenon have long been debated. 

In particular, questions have been raised regarding the interpretation that Muse cells can transdifferentiate into cells that belong to lineages other than mesodermal ones because some groups have suggested that transdifferentiation is a result of fusion between the infused cells and host cells [39,40]. Fusion *in vivo* is indeed conceivable. On the other hand, based on the frequency and ratio of MSCs integrated and differentiated into the host tissue, fusion alone cannot explain all of the phenomena observed after MSC infusion. Furthermore, experiments using a sophisticated Cre-lox system clearly demonstrated the differentiation ability of MSCs into epithelial cells *in vivo* without fusion [41], so a small subpopulation of MSCs is still assumed to be responsible for spontaneous differentiation across the lineage *in vivo*. 

**Figure 4:**
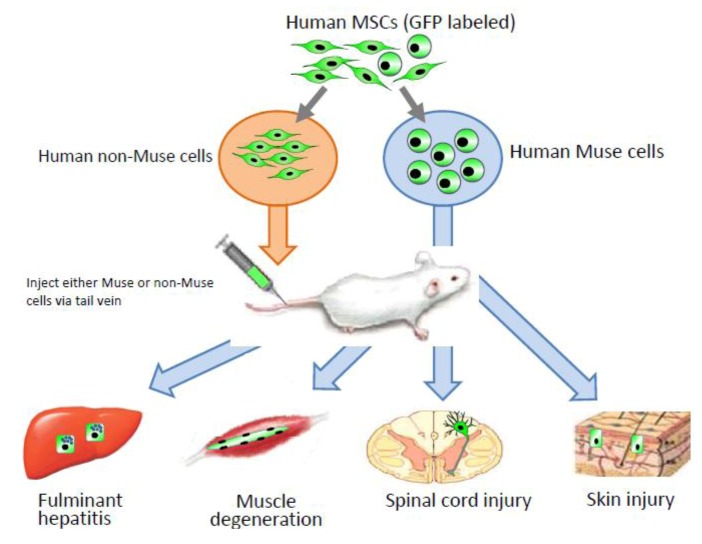
Contribution of Muse cells to tissue repair.

**Figure 5:**
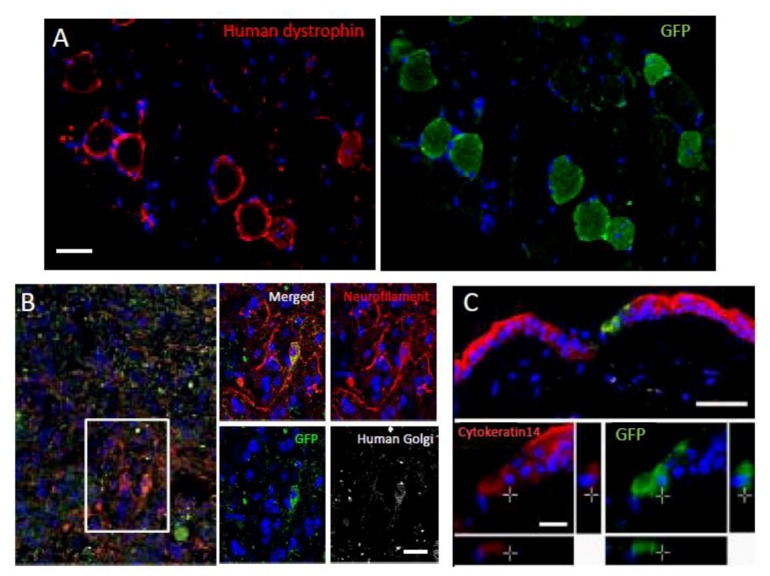
Differentiation and repair effects of Muse cells-1.

**Figure 6:**
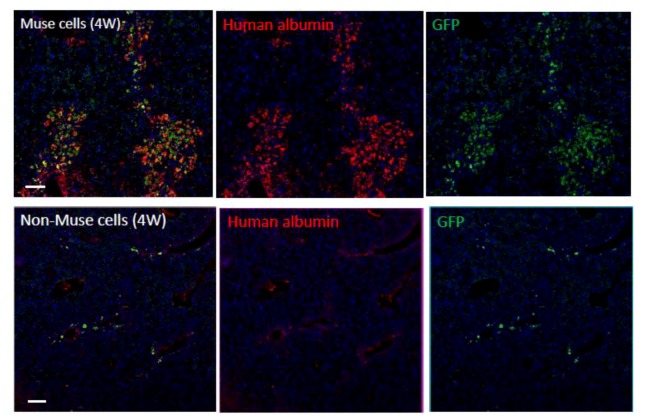
Differentiation and repair effects of Muse cells-2.

Interestingly, without induction or differentiation, naive Muse cells act as ‘repair cells’ when infused into the peripheral bloodstream in acute injury model animals. This was verified by the infusion of green fluorescent protein [GFP]-labeled naive human Muse cells into immunodeficient mouse models with spinal cord injury, skeletal muscle degeneration, skin injury, or fulminant hepatitis (Figure 4). The infused Muse cells homed into damaged sites and differentiated into skeletal muscle cells (human dystrophin-positive; Figure 5A), neuronal cells (neurofilament-positive cells; Figure 5B), keratinocytes (cytokeratin 14-positive; Figure 5C), and hepatocytes (human albumin-positive and human anti-trypsin-positive cells; Figure 6) according to the integrated tissues [3,20]. Some Muse cells were trapped in the lung and spleen, but, interestingly, the majority of Muse cells integrated into damaged tissues and not into intact tissues. Therefore, Muse cells are perceptive of damaged sites perhaps by signals produced by damaged tissues and/or disruption of vessels. The results revealed that Muse cells can integrate as functional cells into damaged tissue and differentiate into ectodermal- (neuronal cell, keratinocytes), endodermal- (hepatocytes), and mesodermal-lineage cells (skeletal muscle cells) according to the site of integration, and contribute to tissue reconstruction. 

Human MSCs were labeled with green flourescent protein (GPF), and then Muse and non-Muse cells were separated. Fluminant hepatitis, muscle degeneration, spinal cord injury, and skin injury models were created in immunodeficient mice that do not reject human cells, and then either Muse or non-Muse cells were infused into the animals by tail vein injection. Local injections were applied only to the skin injury model (Figure 4). Only Muse cells integrated into the damaged tissue, differentiated, and repaired the tissue, while non-Muse cells showed no such phenomena. 

Green fluorescent protein (GFP)-positive Muse cells integrated into muscle degeneration (A), spinal cord injury (B; made by crush injury), and skin injury (C) models, and became human dystrophin- (A), neurofilament- (B; cells were also positive for the human cell marker, anti-human Golgi complex, confirming that the positive cells were of human origin), and cytokeratin 14- (C) positive cells 4 weeks after injection. (Pictures adapted with permission from Y. Kuroda et al. (2010). 2010 The National Academy of Science.) [20].

When Muse cells are separated from MSCs prior to infusion, non-Muse cells, unlike Muse cells, do not integrate or differentiate into functional cells in any of the above-mentioned injury models [20]. Thus, among MSCs, only Muse cells recognize and integrate into the injured site *in vivo*, and differentiate into tissue-specific cells according to the integrated tissue, whereas cells other than Muse cells do not. This also suggests, at least in part, that the tissue repair effects observed following MSC infusion or transplantation are due to Muse cells, whereas non-Muse cells may have trophic and anti-inflammatory effects that contribute to tissue repair in collaboration with Muse cells. 

Some infused Muse cells are trapped in the lung and spleen blood capillaries at 4 weeks and are not detected in intact organs and tissues. For example, in fulminant hepatitis, the vast majority of Muse cells are integrated into the liver, but they are not detected in intact tissues such as the kidney, brain, heart, skeletal muscles, and skin. Disruption of blood vessels and destruction of tissues might be required for naive Muse cells to recognize damage sites and home to repair tissues in the acute phase. Muse cells differentiate into tissue-specific cells according to the integrated tissue, but the factors that define ‘the theory of site’, which instruct Muse cells to differentiate appropriately, are unclear. 

In summary: 

Muse cells are pluripotent stem cells that are able to differentiate into mesodermal-, ectodermal-, and endodermal-lineage cells without exogenous gene introduction and can be directly collected from human tissues. 

Muse cells can be obtained from easily accessed tissues, such as the skin and bone marrow, and from commercially available cultured fibroblasts and BM-MSCs.

Muse cells are non-tumorigenic.

Muse cells correspond to 0.03% of bone marrow mononucleated cells, and ~1% of cultured fibroblasts and BM-MSCs. They are part of MSCs that are already used in the clinic, therefore Muse cells are highly expected to be safe for clinical use.

Muse cells have a proliferation speed of ~1.3 d/cell, slightly slower than that of fibroblasts in adherent culture, so a large number of Muse cells can be prepared.

Muse cells act as repair cells *in vivo.*

## 10. Potential of Muse Cells in Regenerative Medicine

Safety is the most important issue in the clinical application of any kind of stem cell treatment and therefore tumorigenicity requires careful robust consideration. Whether artificially established cells (*i.e.*, ES cell and iPS cells) or cells in a very different developmental stage (fetal stem cells) can truly be integrated into already established adult tissues and whether those cells are able to relate to the surrounding functioning adult cells are issues that must be carefully evaluated. Considering the purpose of regenerative medicine, infused cells must become functioning members of the adult tissue in the fullest sense. Otherwise, transplanted cells will remain unrelated and unconnected cells in adult tissues, such as adhesive plaster. Regarding this point, adult cells may be better suited for treating adult tissues. 

MSCs are currently applied in therapies for patients based on their efficacy in animal models, but their actual features remain poorly understood. Because Muse cells are newly identified in MSCs, MSC therapies with high efficacy might be realized by the appropriate use of Muse cells. For this, it is critical to continue basic research and preclinical studies on Muse cells.

MSCs exert trophic and repair effects and have been applied therapeutically with various kinds of diseases for tissue regeneration and functional restoration [4,5]. The scientific basis for the repair effect of MSCs, however, has not been clearly elucidated. Muse cells are characterized as pluripotent stem cells with a broad spectrum of differentiation ability, and they have been identified as the cells among MSCs that exert repair effects on various kinds of organs and tissues. 

An important issue to be considered carefully for Muse cell treatment is whether Muse cells are the only cells necessary for repair, and whether non-Muse cells are necessary. The major action of non-Muse cells, namely, trophic and anti-inflammatory effects, would not be long-lasting because most MSCs infused as naive cells do not integrate into tissues and are eventually eliminated by phagocytic cells [42]. If the purpose is to repair or regenerate tissues, the use of Muse cells would be reasonable because of their pluripotency and repair effects. In this regard, Muse cells are key cells for treatment, and thus Muse cell-enriched MSCs, *i.e.*, Muse and non-Muse cells mixed in a certain ratio would be practical strategies for treatment. 

Most diseases, however, are a complex of several phenomena and do not comprise a simple pathology. In such cases, a single approach might not be effective to cure the disease. For example, the acute phase of tissue damage, such as acute myocardial infarction or hepatitis, involves tissue inflammation and apoptosis or degeneration of damaged cells. Even though Muse cells are resistant to cellular stress, they may not reach their maximal potential in the tissues during the acute phase because some of them might be damaged before their regenerative effects could be exerted. This would of course decrease the efficiency of cell therapy, and from this standpoint, the best ratio of Muse and non-Muse cells for each disease requires further investigation. Besides, the interactions and cross-talk between Muse and non-Muse cells are other important issues requiring further elucidation.

## 11. Conclusions

Mesenchymal stem cells **(**MSCs) derived from bone marrow, fat tissue, dermis, and the umbilical cord are useful for cell-based therapy in humans because of their low risk of tumorigenesis and easy accessibility [1,2]. MSCs are known to have pleiotropic actions; not only do they exert trophic and anti-inflammatory effects on damaged tissues by producing a variety of trophic factors and cytokines, they also modulate immunologic reactions, which is the basis for their application in graft-versus-host disease [3,4,5]. MSCs that have long been debated to have pluripotency, because they show spontaneous differentiation into mesodermal, ectodermal, or endodermal cells with a very low frequency and are known to home to the damaged site and contribute to tissue repair. Recently, we have found pluripotent stem cells, Muse cells, that comprise ~1% of cultured MSCs and 0.03% of human bone marrow mononucleated cells show self-renewal, triploblastic differentiation and tissue repair effect. Thus, pluripoptency of MSCs may be explained by Muse cells. Importantly, Muse cells do not form tumors when transplanted, so that they are beneficial for clinical application. Besides, MSCs other than Muse cells, namely non-Muse cells, are known to have trophic, anti-inflammatory and immunosuppression effects. Therefore, Muse and non-Muse cells mixed in a certain ratio would be a practical strategy for the treatment of some of diseases.

Green fluorescent protein (GFP)-positive human Muse or non-Muse cells derived from fibroblasts were infused into the tail vein of animals with fulminant hepatitis (4 weeks). Many Muse cells (GFP-positive) integrated into the damaged liver and expressed human albumin, whereas the majority of non-Muse cells did not remain in the liver nor express human albumin. (Figure 6 pictures adapted with permission from Y. Kuroda *et al*. (2010). 2010 The National Academy of Science, and with permission from Wakao *et al*. (2011). 2011 The National Academy of Science. ) [3,20].
